# LC-REHAB: randomised trial assessing the effect of a new patient education method - learning and coping strategies – in cardiac rehabilitation

**DOI:** 10.1186/1471-2261-14-186

**Published:** 2014-12-13

**Authors:** Vibeke Lynggaard, Ole May, Alison Beauchamp, Claus Vinther Nielsen, Inge Wittrup

**Affiliations:** Regional Hospital West Jutland, Cardiovascular Research Unit, Herning, Denmark; Deakin University, Public Health Innovation, Melbourne, Australia; Department of Public Health, Section of Social Medicine and Rehabilitation, Aarhus University, Aarhus, Denmark; CFK - Public Health and Quality Improvement, Central Denmark Region, Aarhus, Denmark

**Keywords:** Cardiovascular disease, Rehabilitation, Patient education, Learning, Coping

## Abstract

**Background:**

Due to improved treatments and ageing population, many countries now report increasing prevalence in rates of ischemic heart disease and heart failure. Cardiac rehabilitation has potential to reduce morbidity and mortality, but not all patients complete. In light of favourable effects of cardiac rehabilitation it is important to develop patient education methods which can enhance adherence to this effective program. The LC-REHAB study aims to compare the effect of a new patient education strategy in cardiac rehabilitation called ‘learning and coping’ to that of standard care. Further, this paper aims to describe the theoretical basis and details of this intervention.

**Methods/design:**

Open parallel randomised controlled trial conducted in three hospital units in Denmark among patients recently discharged with ischemic heart disease or heart failure. Patients are allocated to either the intervention group with learning and coping strategies incorporated into standard care in cardiac rehabilitation or the control group who receive the usual cardiac rehabilitation program. Learning and coping consists of two individual clarifying interviews, participation of experienced patients as educators together with health professionals and theory based, situated and inductive teaching. Usual care in cardiac rehabilitation is characterised by a structured deductive teaching style with use of identical pre-written slides in all hospital units. In both groups, cardiac rehabilitation consists of training three times a week and education once a week over eight weeks. The primary outcomes are adherence to cardiac rehabilitation, morbidity and mortality, while secondary outcomes are quality of life (SF-12, Health education impact questionnaire and Major Depression Inventory) and lifestyle and risk factors (Body Mass Index, waist circumference, blood pressure, exercise work capacity, lipid profile and DXA-scan). Data collection occurs four times; at baseline, at immediate completion of cardiac rehabilitation, and at three months and three years after the finished program.

**Discussion:**

It is expected that learning and coping incorporated in cardiac rehabilitation will improve adherence in cardiac rehabilitation and may decrease morbidity and mortality. By describing learning and coping strategies the study aims to provide knowledge that can contribute to an increased transparency in patient education in cardiac rehabilitation.

**Trial registration:**

Identifier NCT01668394.

## Background

Ischemic heart disease (IHD) and heart failure (HF) are among the leading causes of death in Europe. Further, these diseases cause chronic morbidity and reduced quality of life for around 300.000 patients in Denmark [1]. In recent decades the incidence of IHD has decreased while that of HF has stabilised. However, the prevalence of both has increased due to better treatments and a changing age composition of the population [2, 3].

Cardiac rehabilitation (CR) is thus of great significance due to its known potential to reduce morbidity and mortality, to improve quality of life and affect risk and lifestyle profiles in a positive direction [4–8]. CR was defined by WHO in 1993 as: *“the sum of activities required to influence favourably the underlying cause of the disease, as well as to ensure the patients the best possible physical, mental and social conditions so that they may by their own efforts preserve, or resume when lost, as normal a place as possible in the life of the community”* [9]. A broader definition of rehabilitation from the World Report on Disability in 2011 is: *“a set of measures that assist individuals who experience, or are likely to experience, disability to achieve and maintain optimal functioning in interaction with their environments”* [10]. This broader definition includes the patient’s environment such as their family and friends, where they live, and their workplace as essential factors in rehabilitation, thereby emphasising the global perspective of the term.

Many patients with IHD and HF do not succeed with lasting lifestyle improvements and only a fraction of the relevant patient group complete CR [2]. In light of the favourable effects of CR it is important to develop patient education strategies which can help patients to improve adherence to CR and make changes towards a healthier lifestyle [11, 12].

While patient education has been variously defined and implemented in different chronic disease management programs, only limited evidence has been found for its effectiveness on adherence to CR and adherence to lifestyle change. Furthermore, insight about optimal content is lacking in many patient education programs and large, high-quality studies with a long term perspective are required [13]. Reviews of patient education in CR suggest interventions such as self-monitoring of activity, action planning, use of lay volunteers and tailored counselling by CR staff to promote adherence are likely to be successful. However, data is limited and further research is needed on interventions targeting patient-identified barriers to be able to make practice recommendations for increasing adherence to CR [14, 15].

A patient education strategy called ‘learning and coping’ (LC) was developed in Norway to address some of these challenges in patient education. LC is a health pedagogical strategy that builds on situated and inductive teaching with high involvement from the participants [16]. Characteristics of LC are that ‘experienced patients’ plan, teach and evaluate, together with health professionals. LC was implemented as a pilot project in group based patient education for patients with diabetes and chronic obstructive lung disease in the municipalities of the western part of Central Denmark Region. In this pilot project an extra dimension was included in the Danish adaptation of LC, namely that group based programs began and finished with individual ‘clarifying’ interviews [17].

A study, referred to as LC-REHAB, was designed to address the limitations of the available research on patient education in CR. The study evaluates the effectiveness of LC incorporated in CR on adherence, morbidity and mortality, quality of life plus lifestyle and risk factors in a parallel group open randomised design. Beyond presenting the LC-REHAB study protocol the aim of this paper is to also describe the theoretical basis and details of the LC strategies.

## Methods/Design

### Design

LC-REHAB was launched in three hospital units in Regional Hospital West Jutland (RHWJ), Denmark as an open randomised parallel group controlled study within which participants are allocated to either the intervention arm (LC strategies incorporated in standard CR) or to the control arm (standard CR). The randomisation is computer generated in a 1:1 ratio stratified for hospital unit, diagnosis (IHD or HF) and gender, alternating in blocks of two to four. According to power calculations, a total of 750 patients were needed to be recruited for the primary analysis. For secondary analysis of body compositions with Dual energy X-ray absorptiometry (DXA) scans, n = 150 patients were needed.

The CR program in both the intervention and control arms lasts for eight weeks, with training sessions three times a week and one education session per week. All sessions last 1.5 hours. In total, there are 24 training sessions and eight education sessions. The education in both arms is divided into eight topics; one topic per week. The topics are: function and symptoms of the heart, lifestyle effects on the development of IHD and HF, emotional reactions, medication, tiredness, the importance of relatives or other networks, importance and types of exercise, and future life with a chronic disease. The topics were elected on a theme day by LC educated health professionals and experienced patients with ‘The Disease Management Program for cardiovascular disease’ from Central Denmark Region as a general framework [18]. The training sessions are conducted by a physiotherapist in the presence of a nurse in case patients become unwell. The sessions consist of both cardio-stress and muscle-strengthening exercises. The education sessions are presented mainly by a nurse except for the topics on exercise and future life which are presented by the physiotherapist. In each arm of the study, patients are supported by the same team of nurse and physiotherapist throughout the eight week period.

**Figure 1:**
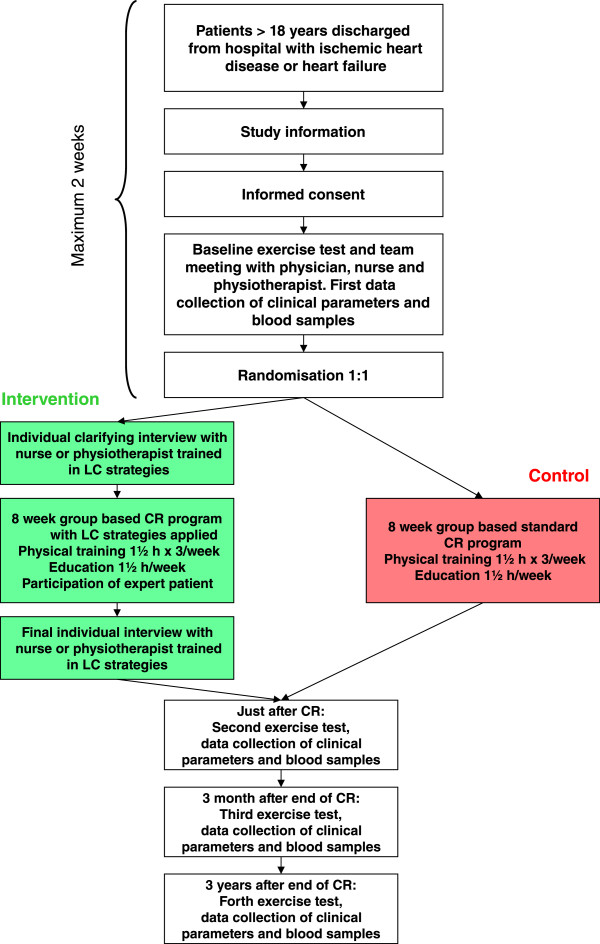
**Flowchart LC-REHAB study.** Cardiac rehabilitation (CR), Learning and Coping (LC).

Data is collected four times: at baseline, just after finishing the eight week CR program, three months after program completion, and at three years after program completion. A bicycle exercise test is conducted at all four data collection points (Figure 1).

Nine CR programs run continuously at the same time in all three hospitals. Eight of these programs are used for the study – four programs for the intervention (LC) arm and four programs for the control arm. The last program is used for patients who do not want to participate in the study. Patients are allocated consecutively to the eight programs which have continuous uptake and a maximum of twelve patients on each program.

### Participants

Recruitment was initiated on 30^th^ of November 2010. Inclusion criteria are: patients aged 18 years and above discharged from hospital with either IHD (ICD-10 codes: I20 and I21) or HF (ICD-10 codes: I50.0-3, I50.8, I50.9, I42.0, I42.6-9, I11.0, I13.0 and I13.2), assigned and motivated for CR. Exclusion criteria are: Acute coronary syndrome less than five days before randomisation, active peri- myo- or endocarditis, symptomatic and untreated valve disease, severe hypertension with blood pressure >200/110 mmHg, other severe cardiac or extra cardiac disease, planned revascularisation, senile dementia, assessed as having low compliance, or former participation in the study.

### Recruitment/settings

Study information is sent by mail to eligible patients and they are informed of the study via telephone by this paper’s first author. Informed consent is obtained one to two weeks after discharge by health professionals working in the CR units; either a nurse or a physiotherapist.

All CR programs start the first working day after randomisation and are performed in the CR units at each of the three hospitals. The majority of measurements are taken by health professionals or via questionnaires. Laboratory testing is performed at the hospitals’ laboratories, and DXA scans are performed at the Department of Nuclear Medicine within two of the three hospitals.

### Group allocation

#### Intervention group

CR programs with incorporated LC strategies start with an individual clarifying interview followed by an eight week group based training and education period and finish with another individual clarifying interview. Experienced patients participate as co-educators in all of the education sessions and in one of the training sessions each week. Each week, an hour long evaluation meeting is conducted by the team of nurse, physiotherapist and experienced patient who are assigned to the specific program.

The LC health professionals - nurses and physiotherapists - have completed a competence-education course in LC strategies lasting eight days with the experienced patients participating in the last four. Public Health and Quality Improvement, Central Denmark Region, Aarhus, Denmark conducts this course containing lectures, group work and supervision. An outline of this course can be obtained from the author. Experienced patients were recruited from participants in previous CR programs. The experienced patients are clinically stable and have a high level of coping with their disease. The latter was determined by this paper’s first author during interview, based on her experience as a cardiology nurse and her knowledge of LC strategies. LC strategies take a situated, reflexive and inductive approach to education, by focusing on the individual patients’ needs and concerns in relation to the present topic rather than following a structured, pre-written slide show. Materials are developed for each topic including background literature and questions designed to facilitate discussion among patients.

### Principles, theoretical basis and practical implementation of LC strategies

#### Understanding the patients’ perceived illness

##### Theory

The use of experienced patients is an innovative dimension in CR. This new approach requires health professionals to face the paradox between two aspects of sickness; ‘disease’ and ‘illness’, as introduced in 1978 by the American psychiatrist and anthropologist Kleinman. In this context, ‘disease’ refers to a malfunction of a biological process while the term ‘illness’ refers to the psychosocial experience and meaning of perceived disease [19]. The English sociologist Bury described chronic illness as a biographical disruption. He linked three aspects of disruption in development of a chronic disease; the disruption of taken-for-granted assumptions, the profound disruptions when the patient emerges from the illness and finally the disruption involving the patient’s mobilisation of resources to face his or her altered situation [20]. Charmaz took ‘biographical disruption’ a step further and called it a loss of self – a fundamental form of suffering in the chronically ill [21]. Disruption as described by Bury is a basic condition, and in order to search for meaning in life it is necessary to address the small routines of everyday life [20]. The individual’s account of the origin of their chronic illness in terms of causes can also, according to Williams, be read as an attempt to establish points of reference between body, self and society in reconstructing a sense of order from the fragmentation produced by chronic illness [22].

##### Practical implementation

When experienced patients tell their narratives about living with a heart condition, it is a way of integrating their perceptions about their illness with the professional’s knowledge about heart disease. The ‘illness’ term described by Kleinman is represented by the experienced patients while the ‘disease’ term is represented by health professionals. In this way, potential gaps in belief about disease between health professionals and patients can be bridged in the mutual ‘teaching room’. To support this, LC trained health professionals consistently focus on the participants’ everyday life during the group-based education and training sessions. They explore the patients’ daily routines as a means to help them bring order to their chaos and feelings of loss. Health professionals also address patients’ everyday lives in the individual clarifying interviews and allow patients to talk about the origin of their disease and their experiences of suddenly being ill.

#### Structured use of narratives

##### Theory

Narratives are described as a way of creating meaning, importance and consistency in the disorder, chaos and uncertainty that chronic illness brings [19, 22–25]. Telling a narrative about one’s illness has a potentially therapeutic effect especially on the narrator and also allows the audience to become involved in the story being told [26, 27]. Narratives are dynamic and in addition to creating order and structure out of past experiences, they give new perspectives to the present and assist in shaping the future. New experiences become meaningful when they are intertwined with existing experiences and existing experiences are reinterpreted and reshaped when they are entangled with new experiences [28]. Sharing brief examples of other, similar patients’ successes in changing a habit or solving a problem could be inspiring [29]. Narratives are not just mirrors of reality but are created in a relationship between the narrator and the audience. The listeners play an active role in the narratives and as such, expand reflections on possibilities for both health professionals and patients [26, 27].

##### Practical implementation

In the LC program, narratives from experienced patients are used as a method to bring experience-based knowledge about heart disease into teaching. These narratives aim to inspire patients to tell their own stories. Teaching is undertaken hand-in-hand with health professionals, who encourage participating patients to contribute their own narratives, providing new perspectives to the stories being told, creating a confident and trustworthy situation. The use of experienced patients and narratives in the LC program are steps in the process towards health behaviour change for participants. The power of having experienced patients who have changed a variety of behaviours during their lifetime assists the health professionals by showing them that participating patients are in fact able to make the necessary changes.

#### Understanding ‘learning’ as an ongoing process

##### Theory

The learning style in the LC program relies on the learning triangle developed by the Danish psychologist Illeris. He defines learning as an ongoing process consisting of two integrated processes of interaction and internalisation respectively, and describes learning as simultaneously comprising three different dimensions; cognitive, emotional and social. Learning and every single learning process reaches across these three dimensions. The ‘emotional drive’ for instance must be present for a learning process to become successful. If patients feel they are at risk, are uncertain, or have an unmet need, this impacts on their ability to learn. If the emotional or social dimension is not covered in a teaching situation the receiver attempts to restore the balance by developing greater sensitivity to themselves or their social environment [30].

##### Practical implementation

The health professionals in the LC program do not impose an ‘emotional drive’ on the patients, but instead try to motivate them to find the drive themselves in order to restore balance to the learning triangle. This helps the patients to understand learning as an ongoing process that they can integrate into the rest of their lives.

#### The appreciative approach

##### Theory

The overall approach in LC strategies is ‘appreciative inquiry’ as introduced by Cooperrider in 1987; a way of exploring patients’ previous experiences of using successful coping strategies earlier in their life. Applying strategies that have worked in the past to the future is a way of visualising the road to get there [31]. The ‘wanted’ future is connected to positive experiences in the past. Visions for the future, which the patients can identify with and which are created through talk, can create possibilities for behavior change instantly [32].

##### Practical implementation

To facilitate the appreciative approach the focus in the LC program is on the individual patient’s resources and successes, although their concerns are also valued. The aim of the initial clarifying interview is to initiate a developmental process in the patients before the rehabilitation program in order for them to achieve a better understanding of their needs, and with that knowledge prepare them for coping more easily in life. This awareness of the patient’s experiences and the building of a relationship provide valuable insights for the health professionals later in the group-based sessions where they can raise issues from the initial clarifying interview (if ethically acceptable). In the second interview at the end of the rehabilitation program, the health professional clarifies with the patient how they had benefited from the group-based sessions, and supports them to identify future strategies for coping with their chronic disease.

#### Motivating patients to change behaviour towards a healthier lifestyle

##### Theory

The individual interviews and the group based sessions are based on the concept of ‘motivational interviewing’ (MI) which has previously been shown to be effective in changing health behaviour, including in CR [33]. MI activates the patient’s own motivation to change health behaviour and adhere to a treatment plan. Motivation for change can be affected, worked with and developed in the context of a personal relationship. The concept relies on four main principles; roll with resistance, develop discrepancy, express empathy and support self-efficacy [29, 34].

##### Practical implementation

The purpose of having the experienced patients participate once a week in the training is to show that even experienced patients have varying exercise capabilities. Participating patients might thus identify with the experienced patients and be motivated to put extra effort into training.

#### Participant involvement by inductive teaching

##### Theory

Health professionals use four different types of questions throughout the LC rehabilitation program; lineal, circular, strategic and reflexive, in order to promote greater involvement from participants. The theoretical basis relies on the psychiatrist Tomm’s definition. He claims that the therapists must take responsibility for the questions they ask in order to increase the therapeutic value of the interview. The approach suggests that a particular question invites a particular answer, and that selecting either a lineal, circular, strategic or reflexive question restrains the range of “legitimate” responses and gives the health professionals influence in maintaining a direction for the conversation [35].

##### Practical implementation

This approach is demonstrated in the the LC training strategies, which aim to encourage participating patients to make exercise a part of their everyday life. The physiotherapist helps patients to identify challenges when exercising outside the hospital. They ask questions such as: Are you afraid when you feel your heart beat increasing? How do you cope with your body’s signals? How can you transfer this to your everyday life? Also the use of personal narratives about how patients manage the exercises at home and any challenges they experience is a central part of the training sessions, and is used to create reflections amongst patients. Health professionals request the patients to tell these stories either in the group-based setting or in a smaller personal context as a therapeutic attempt to bring order to their concerns and allay any anxiety about performing cardio stress exercises.

#### Homework for patients between sessions

##### Theory

Allowing time between the CR sessions is an important opportunity for the patients to achieve learning and coping skills that can eventually enhance their self-efficacy. The Danish and American anthropologists Grøn, Meinert and Mattingly define the concept ‘chronic homework’ as work that patients and families are expected to carry out in their home in order to manage their chronic condition. However, this chronic homework might look very different from the perspectives of health professionals and those of the patients. In their home, patients are unconsciously controlled by bodily habits and routines and it is thus important for health professionals to avoid the homework - as defined by the expert health sector - colonising the patient’s private sector. Therefore, the authors argue the need to look at chronic homework as a partnership that health professionals and patients must create with one another in a common understanding; a ‘borderland practice’ that moves between home and clinic, and is in continual need of reinvention as lives and home situations change [36].

##### Practical implementation

In the LC program, patients participate in defining their own homework between the CR sessions and health professionals help patients to integrate their acquired health-knowledge and concerns into their everyday lives.

#### Teaching the patients about stages of coping

##### Theory

Change is a process that involves progression through a series of different stages. This was defined in the theory ‘stages of change’ and contains the concepts: precontemplation, contemplation, preparation, action, maintenance and termination. Progress through the stages is not a linear process but consists of a spiral pattern whereby relapses to former stages can occur before reaching the maintenance or termination stage [37, 38]. For behaviour change to succeed and to build self-efficacy, people must feel threatened by their current behavioural patterns and believe that change of a specific kind will result in a valued outcome at an acceptable cost [39, 40]. Different ways of coping may influence behaviour change, and psychological and physical health outcomes [41].

##### Practical implementation

Different ways of coping are reviewed during the LC education sessions. An illustration of a spiral-formed coping circle is used in all group-based education sessions so patients can identify their stage of coping [42]. In that context, the health professionals emphasise to patients that healthy behaviour changes do not occur overnight but must be worked upon continuously.

### Evaluation meetings

The same team of nurse, physiotherapist and experienced patient conduct all the sessions during a period of eight week LC program. This reflective team evaluates the previous week in an hour-long meeting in which they discuss the overall sessions and needs of individual participants. The team evaluates what went well and what was less successful. They identify which experienced patient narratives worked as good learning examples in the ‘teaching room’ and discuss how health professionals can elaborate the narratives and make them generally applicable. In this scheduled evaluation, the team also makes plans for the following week’s education and training sessions.

### Summary of the intervention

Experience-based knowledge about heart diseases is integrated into LC CR via experienced patients as ‘bridge builders’ telling their narratives about how life can unfold when living with a chronic heart condition. Health professionals continuously address patients’ everyday lives in order to build partnerships in the ‘borderland’ between home and clinic. The educational tools rely on Illeris’ learning triangle and motivational interviewing whereby the health professional focuses on the theories of coping, ‘stages of change’ and ‘self-efficacy’. The LC strategies are tools to help health professionals understand how body, self and society interact and may contribute to building partnerships between patients and health professionals within their different contexts.

### Control group

Standard CR is characterised by a structured deductive teaching style with use of an identical pre-written slide show at all three hospital units. The eight week program is group based and the health professionals performing the training and education sessions are recruited from the cardiology wards and the departments of physiotherapy, respectively. They have not been given any formal education in CR but have achieved their skills by sitting in on sessions until they felt they were able to carry out the training and education themselves.

### Outcomes

The primary outcomes are adherence to CR, morbidity and mortality. Secondary outcomes are quality of life as well as lifestyle and risk factors. Socioeconomic variables and other relevant characteristics are collected at baseline.

Adherence to CR is assessed as completing the CR program, defined as having performed the second exercise bicycle test at the end of the program as well as determining the proportion of attended versus planned training and education sessions. Morbidity and mortality data will be drawn from the Danish National Patient Registry and the Danish CPR-registry. All readmissions after randomisation regardless of diagnosis will be included as the intervention is aiming more broadly than only heart disease-related events. Readmissions for IHD or HF as a sub-measure will also be assessed. Survival is measured as a combined endpoint of death, readmission with acute coronary syndrome, revascularisation or unstable angina. Data on medication compliance will be drawn from the Danish national medication profile as an independent variable.

Quality of life is measured with Short Form-12, version 2 (SF-12) questionnaire [43]. Furthermore, SF-6D is measured; this is a preference based measure of health derived from the SF-12 and often used as quality-adjusted life years (QALY) [44]. The Health education Impact questionnaire, version 3 is used to measure the patients’ coping ability. This is a questionnaire with high validity and reliability developed in Australia to evaluate the impact of patient education programs [45, 46]. Depression is assessed with the Major Depression Inventory. This questionnaire is validated in a Danish trial and has good accordance with depression diagnosis made by a psychiatrist [47].

Self-reported lifestyle and risk factors are measured with questionnaires on diet, smoking and exercise. Weight, height and waist circumference are measured by health professionals at all data collection times. Body composition of fat, muscle and bone are determined by DXA scans. Blood pressure is measured in a sitting position after 5 minutes of rest. Exercise work capacity is estimated from a maximum symptom-limited exercise test on a bicycle ergometer starting with 25 watts, adding 25 watts every 2 minutes. Lipid profiles include fasting triglycerides, total cholesterol, LDL-cholesterol and HDL-cholesterol.

### Power calculations

From an existing CR database in RHWJ the standard deviation (sd) on the change in the SF-12 scales was known. It is expected that better coping ability could affect the scales ‘vitality’ (sd = 24.4 percentage points) and ‘mental health’ (sd = 13.4 percentage points). Necessary participant numbers were calculated under different assumptions which led us to choose a sample size of minimum 750 patients. If all patients complete the program, a difference in vitality of 5 percentage points and a difference in mental health of 2.75 percentage points will be detected with a power of 80% and a p-value of 0.05. With a dropout of 10 and 20% respectively, a difference in vitality of 5.3 and 5.6 respectively, and a difference in mental health of 2.9 and 3.1 respectively, will be found under the same assumptions.

From in vivo precision of DXA scans a sd of 0.187 in total body fat (TBF) at two consecutive measures was known [48]. We will include at least 150 patients to receive DXA scans in this study. Selection was based on the computer generated randomisation, thus every fourth participant was allocated for DXA scan – stratified for two hospital units, gender and diagnosis. If all 150 patients attend the DXA scan a difference of 0.09 kg in TBF will be able to be shown with a power of 80% and a p-value of 0.05.

### Statistical methods

Data is entered into dedicated databases and statistical analysis will be performed using STATA13 [49]. Cleaning and data management will be carried out on all relevant datasets and all statistical analysis will be performed using the ‘intention to treat’ principle. Descriptive statistics will be used to describe baseline variables. Differences in change in mean scores from baseline to follow-up between the two groups for continuous variables will be tested using unpaired t-tests provided they are normally distributed. Non-parametric tests will be performed for non-normally distributed data. Multiple regression analysis will be undertaken to check for interactions between gender, age and socio-economic background. For dichotomous variables, chi-square tests or logistic regression will be performed. Readmissions and survival will be analysed using Cox regression analysis.

### Ethical considerations

The intervention carries no risks and all patients will have at least standard treatment. The participants will only be included in the study, if they give written consent after verbal and written information. All patients will participate on a voluntary basis and will be informed that they at any time can withdraw their informed consent. The study will be carried out in agreement with the Declaration of Helsinki II and in accordance with Good Clinical Practice. The study has been approved by the Ethical Committee of Central Denmark Region (journal number 20100230).

## Discussion

In the future, increasing numbers of people will have to live with a chronic heart condition. Cardiac rehabilitation is thus of great significance due to its known potential to reduce morbidity and mortality and improve quality of life. Different patient education methods have been developed to address the challenges of non-adherence to CR programs and limited success with lasting lifestyle improvements. In the LC-REHAB study, the effect of a new patient education strategy; learning and coping, in addition to standard CR is evaluated on morbidity, mortality, adherence, quality of life plus lifestyle and risk factors. To understand the study design and rationale more comprehensively, knowledge of LC and the theory underlying its strategies is required. This paper combines a description of LC strategies and their theoretical basis along with the LC-REHAB study protocol.

Patients are followed up to three years after they have finished CR. This will provide insight into the long-term effect of CR and especially the effect of the LC strategies. The study is un-blinded. Combined with the self-reported information of quality of life, diet, smoking and exercise this may lead to differential misclassification.

This paper aims to provide knowledge and transparency of the theories behind LC strategies. The hypothesis is that LC strategies applied in CR can enhance adherence in CR and thus lead to reduced morbidity, morbidity and readmissions, and improve quality of life for patients with IHD or HF. Additionally, the results of the study will be a significant contribution to the development of relevant evidence-based guidelines for effective CR.

## Abbreviations

CPR: Central person register
CR: Cardiac rehabilitation
DXA: Dual energy X-ray absorptiometry
HDL-cholesterol: High-density lipoprotein cholesterol
HF: Heart failure
IHD: Ischemic heart disease
LC: Learning and coping
LDL-cholesterol: Low-density lipoprotein cholesterol
MI: Motivational interviewing
QALY: Quality-adjusted life years
RHWJ: Regional Hospital West Jutland
SD: Standard deviation
SF-12: Short form-12
TBF: Total body fat
WHO: World Health Organization.
